# Editorial: Effect of mechanical loading on the tendon for tissue engineering approaches

**DOI:** 10.3389/fbioe.2025.1728779

**Published:** 2025-11-06

**Authors:** Clemens Gögele, Herbert Tempfer, Mersedeh Tohidnezhad, Florien Jenner, Denitsa Docheva

**Affiliations:** 1 Institute of Anatomy and Cell Biology, Paracelsus Medical University, Nuremberg, Germany; 2 Institute of Tendon and Bone Regeneration, Paracelsus Medical University Salzburg, Salzburg, Austria; 3 Austrian Cluster for Tissue Regeneration, Vienna, Austria; 4 Faculty of Medicine, Health and Medical University, Krefeld, Germany; 5 Veterinary Tissue Engineering and Regenerative Medicine Vienna (VETERM), Equine Surgery Unit, University of Veterinary Medicine Vienna, Vienna, Austria; 6 Department of Musculoskeletal Tissue Regeneration, Orthopaedic Hospital König-Ludwig-Haus, University of Würzburg, Würzburg, Germany

**Keywords:** tendon, mechanical loading, mechanostimulation, Mechanosensation, biofabrication, *in vitro* and *in vivo* models, inflammation

## Introduction

The field of tendon repair and engineering stands at a pivotal crossroads. Decades of descriptive biology and biomechanics have demonstrated that tendon cells and matrix are exquisitely load-sensitive (Wang, 2006). However, the translation of this knowledge into clinically robust, load-competent grafts remains limited (Szczesny and Corr, 2023; Wang et al., 2025). This translational gap reflects more than just technological inertia—it arises from the inherent complexity of replicating the dynamic mechanical environment that tendons experience *in vivo* (Freedman et al., 2018). Most current biofabrication systems still rely on static culture conditions, lacking the dynamic, cyclic and multidirectional forces that characterize the native tendon environment (Mirsky et al., 2024; Sander et al., 2022). Consequently, even sophisticated constructs often fail to acquire the hierarchical alignment and resilience required for physiological function (Chen et al., 2025; Li et al., 2023). Given the impact of magnitude, frequency, direction, and duration of loading on cell fate and matrix organization, defining and reproducing these mechanical parameters is essential for guiding tenocyte behavior and tenogenic matrix assembly (Benage et al., 2022; Wang et al., 2012). These mechanical variables act through defined mechanotransduction pathways, which in turn regulate inflammation, differentiation, and remodeling—making them actionable targets for therapeutic and bioengineering strategies (Lavagnino et al., 2015; Wang et al., 2018).

The papers included in the Frontiers Research topic *“Effect of mechanical loading on the tendon for tissue engineering*,” collectively update and refine the agenda in four ways: (1) mechanobiological principles should be mapped to biofabrication workflows; (2) there is a need for smarter *in vivo* models and integration of AI and 3D-bioprinting; (3), strain-dependent inflammatory and fibrotic signaling must be elucidated in human tendon and ligament cells; and (4), tissue engineering benchmarks must be grounded in *in vivo* loading metrics should be proposed. Together, these studies argue for an integrated translational pipeline that couples physiology-inspired loading, immune-aware constructs, and rigorous functionally-relevant preclinical metrics.

A short synopsis of each article published in this Research Topic is given bellow:

Gögele et al. propose the central hypothesis that mechanostimulation-guided biofabrication can yield structurally and functionally superior tendon constructs. The authors concluded that successful translation of tendon mechanobiology into biofabrication requires scaffolds and bioreactors that mimic physiological cyclic stretch, frequency, and anisotropic cues that drive tenogenic differentiation and hierarchical matrix assembly. Their review synthesizes mechanosensitive pathways, cell–matrix feedback loops, and examples of cyclic-stretch regimens, arguing that precise, tunable mechanostimulation should be a core design parameter of any tendon biofabrication platform. Without incorporating dynamic mechanical loading (not solely biochemical cues), engineered tendons will inevitably fail to recapitulate the mechanics and function of native tissue (Gögele et al.).

Aykora et al. propose the systems-level integration of harmonized *in vivo* models coupled with artificial intelligence (AI) and three-dimensional bioprinting to reduce the current gap between research and translation and expedite clinically significant tendon regeneration. They contend that current preclinical paradigms are fragmentated, and often limited by inconsistent loading conditions and poorly standardized endpoints. By contrast, AI-assisted analytics applied to standardized models can extract mechanophenotypes from multimodal datasets, while 3D bioprinting will provide sophisticated control over spatial cell–matrix architecture, including tendon/ligament-like tissues. The integration of modeling, computation and fabrication offers a path beyond incremental, device-centric development toward adaptive data-driven tissue engineering (Aykora et al.).

Heidenberger et al. demonstrate that ligamentocytes’ response to mechanical strain is context-dependent, shaped by both the magnitude of loading and the surrounding biochemical environment. Physiologic dynamic strain can attenuate pro-inflammatory and profibrotic signaling, whereas excessive strain promotes inflammation and maladaptive remodeling. Moreover, the transcriptional and matrix responses to strain are modulated by cytokine context, underscoring that mechanical and biochemical cues interact rather than act in isolation. Together, these findings establish that mechanotransduction is not a passive background process but an active determinant of ligamentocyte fate. Consequently, anti-inflammatory or anti-fibrotic strategies that disregard the mechanical context may prove ineffective or even counterproductive (Heidenberger et al.).

Muscat and Nichols argue that *in vivo* tendon loading metrics should define success criteria for engineered constructs. Their review describes animal models of tendon loading and compiles reproducible mechanical and structural readouts, including strain magnitudes, loading regimens, and extracellular matrix organisation, that correlate with functional recovery in animal models. The authors emphasize that isolated molecular markers or single tensile tests are insufficient; engineered constructs must be assessed against the same loading performance and criteria for matrix alignment, stiffness, fatigue resistance and biologic integration expected of native tendons. The calls for physiologic benchmarking and preclinical pipelines that test engineered tissues under loading regimes that mirror the target biology, sets a new translational standard for tendon tissue-engineering (Muscat and Nichols).

## Concluding perspective — toward a mechanobiology-based translational pipeline

Taken together, the papers in this Research Topic map a rational research roadmap for the next phase of tendon research and engineering: i: embed physiologic mechanostimulation into biofabrication approaches, ii: integrate AI and advanced printing to produce engineered, multiscale constructs, iii: account for immuno-mechanical crosstalk and strain-dependent cytokine responses in therapeutic development, and iv: benchmark engineered tissues against *in vivo*-derived mechanical and structural metrics.

The next frontier is not conceptual but operational: To ensure that engineered tendons can meet the demands exerted upon native tissues, the field must standardize loading protocols, share open datasets, and couple mechanobiology with immune modulation and high-fidelity preclinical testing. Only through such integration can we ensure that engineered tendons are not merely biological imitations but functional, mechanocompetent tissues capable of enduring the demands of life.
